# Excessive cleavage of von Willebrand factor multimers by ADAMTS13 may predict the progression of transplant-associated thrombotic microangiopathy

**DOI:** 10.1016/j.rpth.2024.102517

**Published:** 2024-07-22

**Authors:** Shinya Yamada, Kazuya Sakai, Masayuki Kubo, Hirokazu Okumura, Hidesaku Asakura, Toshihiro Miyamoto, Masanori Matsumoto

**Affiliations:** 1 Department of Hematology, Kanazawa University, Kanazawa City, Ishikawa, Japan; 2 Department of Blood Transfusion Medicine, Nara Medical University, Kashihara City, Nara, Japan; 3 Department of Hematology, Toyama Prefectural Central Hospital, Toyama City, Toyama, Japan; 4 Department of Hematology, Nara Medical University, Kashihara City, Nara, Japan

**Keywords:** ADAMTS13 protein, hematopoietic stem cell transplantation, proteolysis, thrombotic microangiopathy, von Willebrand factor

## Abstract

**Background:**

Transplant-associated thrombotic microangiopathy (TA-TMA) is a fatal complication of hematopoietic stem cell transplantation and is characterized by severe thrombocytopenia, hemolytic anemia, and organ dysfunction. In response to several possible triggers, dynamic multimetric change in von Willebrand factor (VWF) may contribute to inducing microthrombi in circulation in TA-TMA.

**Objectives:**

By performing VWF multimer analysis and measuring VWF-degradation product (DP), we unraveled the relationship between multimeric changes in circulating VWF and the pathogenesis of TA-TMA.

**Methods:**

This study analyzed 135 plasma samples from 14 patients who underwent allogeneic hematopoietic stem cell transplantation at a single institute. VWF-associated markers, namely VWF:antigen (VWF:Ag), VWF-DP/VWF:Ag ratio, VWF:ristocetin cofactor activity, VWF:ristocetin cofactor activity/VWF:Ag ratio, and ADAMTS13 activity, were analyzed in these samples collected every 7 days.

**Results:**

There were 2 patients with definite thrombotic microangiopathy (TMA) and 6 patients who presented with probable TMA that did not progress to definite TMA. Each plasma sample was classified into 3 groups: definite TMA, probable TMA, and non-TMA. VWF multimer analysis showed the absence of high-molecular-weight VWF multimers in probable TMA, whereas the appearance of unusually large VWF multimers was observed in definite TMA. The median value of the VWF-DP/VWF:Ag ratio in probable TMA was elevated to 4.17, suggesting that excessive cleavage of VWF multimers by VWF cleaving enzyme, ADAMTS13, resulted in the loss of high-molecular-weight VWF multimers.

**Conclusion:**

During the transition from probable to definite TMA, drastic VWF multimer changes imply a switch from bleeding to thrombotic tendencies. Extensive VWF-DP and VWF multimer analyses provided novel insights.

## Essentials

• Transplant-associated thrombotic microangiopathy (TA-TMA) is a fatal thrombotic complication.
• von Willebrand factor (VWF) multimer and its degradation product were studied in a small cohort.
• The loss of high-molecular-weight VWF multimer was observed just prior to the onset of TA-TMA.
• The excessive cleavage of VWF multimers by ADAMTS13 may trigger the development of TA-TMA.

## 1 Introduction

Transplant-associated thrombotic microangiopathy (TA-TMA) occurs after hematopoietic stem cell transplantation (HSCT) and presents as microangiopathic hemolytic anemia, consumptive thrombocytopenia, and organ dysfunction due to microcirculatory insufficiency [1,2]. Various factors, such as conditioning therapy, immunosuppressive drugs, and allogeneic immune responses, cause vascular endothelial cell damage and platelet thrombus formation [3]. With various diagnostic criteria for TA-TMA [[4], [5], [6], [7]], the incidence and mortality rates of TA-TMA in allogeneic HSCT are 10% to 25% [8] and 60% to 75% at 3 months, respectively [4,9].

von Willebrand factor (VWF) is a hemostatic glycoprotein produced in endothelial cells that is secreted as an unusually large (UL) multimer during vascular injury [10]. UL-VWF multimers can be cleaved into smaller multimers by a disintegrin-like metalloproteinase with thrombospondin type 1 motif 13 (ADAMTS13) at the peptide bond between Tyr1605-Met1606 of the VWF A2 domain [11], thereby preventing excessive platelet aggregation. Since larger VWF multimers possess higher platelet aggregation capacity [12], insufficient cleavage of UL-VWF multimers due to a deficiency in ADAMTS13 activity results in lethal thrombosis in patients with thrombotic thrombocytopenic purpura (TTP). In contrast, there are conditions under which high-molecular-weight (HMW)-VWF multimers are reduced or absent. There are 2 types of VWF abnormalities: von Willebrand disease type 2A [13] and 2B [14], in which the production of HMW-VWF multimers is reduced or consumptively reduced, and acquired von Willebrand syndrome. Aortic stenosis [15], left ventricular assist devices [16], percutaneous cardiopulmonary support [17], and extracorporeal membrane oxygenation [18] are some of the conditions that can cause acquired von Willebrand syndrome and severe bleeding symptoms.

There have been several reports on the association of VWF and ADAMTS13 with complications of allogeneic HSCT; an elevated VWF:antigen (VWF:Ag; ≥325%) is a predictive marker for the development of TA-TMA, and an elevation of VWF:Ag before 37 days (IQR, 20-125 days) of the TA-TMA leads to a definitive diagnosis [19]. An increase in VWF:Ag levels on day 7 after HSCT was a predictive marker of the onset of TA-TMA [20]. All these reports have investigated the relationship between VWF and pathologic conditions involving endothelial damage and microcirculatory disturbance. A VWF-degradation product (DP) enzyme-linked immunosorbent assay (ELISA) was recently developed in our laboratory to quantify the amount of cleavage of VWF multimers by ADAMTS13. The measurement of VWF-DP explains the mechanism of VWF multimer changes. Little is known about the behavior of VWF-DP and VWF multimer patterns in patients with TA-TMA. Thus, this study investigated the association between temporal changes in VWF-associated biomarkers, including VWF-DP, in patients with and without TA-TMA.

## 2 Methods

### 2.1 Patients and blood sampling

Japanese patients aged 20 years or older who underwent allogeneic HSCT for hematopoietic malignancies between June 2020 and March 2021 at the Department of Hematology, Toyama Prefectural Central Hospital, were included. Peripheral whole blood was collected with sodium citrate as an anticoagulant every 7 days, starting on day −7 until a maximum of 63 days or until discharge from the hospital, with the day of allogeneic HSCT as day 0. However, deviations of up to ±2 days were allowed at each blood collection point. All samples were stored at −80 °C, thawed at 37 °C immediately before testing, and were not refrozen.

The study protocol complied with the ethical guidelines of the Declaration of Helsinki and was approved by the Ethics Committees of Nara Medical University (#2442) and Toyama Prefectural Central Hospital (#58-62). Written informed consent was obtained from all the participants.

### 2.2 Diagnosis of TA-TMA

The diagnosis of TA-TMA is based on the Blood and Marrow Transplant Clinical Trial Network (BMT-CTN) criteria [4]: (1) red blood cell fragmentation and ≥2 schistocytes per high-power field on peripheral smear, (2) concurrent increased serum lactate dehydrogenase level above institutional baseline, (3) concurrent renal and/or neurologic dysfunction without other explanation, and (4) negative direct and indirect Coombs test results. In this study, “definite thrombotic microangiopathy (TMA)” was defined when all 4 BMT-CTN criteria were met. “Probable TMA” was defined as TMA that met all of the BMT-CTN criteria except for concurrent renal and/or neurologic dysfunction without other explanations. The test results at each blood sampling point were used to determine the probability of a patient having a TMA occurrence. Based on the test results at each blood collection point, we determined whether each sample could be classified as non-TMA, probable TMA, or definite TMA.

### 2.3 Measurements of VWF-associated biomarkers

Protocols for the measurement of VWF-associated biomarkers have been described elsewhere. Briefly, VWF:Ag levels were measured by sandwich ELISA using rabbit antihuman VWF polyclonal antibodies (Dako Cytomation) [21]. VWF:ristocetin cofactor (RCo) measurements were performed on a CS analyzer (Sysmex UK Ltd) using the BC von Willebrand Reagent (Siemens Healthineers). ADAMTS13 activity levels were measured via a chromogenic ADAMTS13-act-ELISA [22] (Kainos Laboratories). Pooled normal plasma was produced from 20 healthy volunteers, and then, the values of 100% VWF:Ag and ADAMTS13 levels were assigned to this pool. The World Health Organization Sixth International Standard for blood coagulation factor VIII and VWF was used as the standard plasma for VWF:RCo measurements. VWF-DP levels were measured using sandwich ELISA with N10, a monoclonal antibody recognizing a decapeptide (1596-DREQAPNLVY-1605) derived from the VWF-A2 domain, and an anti-VWF monoclonal antibody recognizing the N-terminal region of VWF, developed in our laboratory [23]. The VWF-DP/VWF:Ag ratio was obtained by dividing the measured VWF-DP in each sample by VWF:Ag.

### 2.4 VWF multimer analysis and VWF multimer index

VWF multimer analysis was performed as previously described [14] with some modifications [24,25]. VWF:Ag level in each sample was not equal; however, to avoid technical errors due to the dilution process, the VWF:Ag was not matched to the same level by dilution. VWF multimer bands were classified into 4 groups and defined as follows: low molecular weight (LMW), including 5 bands at the bottom; intermediate molecular weight with the next 5 bands; HMW with upper bands above 10 bands; UL, which is not found in healthy individuals.

The VWF multimer index was measured according to the method described by Tamura et al. [26]. Using ImageJ software (National Institutes of Health), the center of the lane was scanned, and the multimers were divided into 3 parts (LMW, intermediate molecular weight, and HMW + UL-VWF multimers). First, the VWF multimer ratio was determined as the ratio of the VWF multimer area of each part to the total VWF multimer area. Next, the VWF multimer ratio of each part in normal plasma, which was electrophoresed simultaneously, was determined. The VWF multimer index was defined as the percentage of the VWF multimer ratio in each part of the sample that corresponded to the VWF multimer ratio in each part of normal plasma (Supplementary Figure).

### 2.5 Statistical analysis

Comparisons among the 3 groups (non-TMA, probable TMA, and definite TMA) were performed using the Kruskal–Wallis test, and comparisons between each group were performed using the Steel–Dwass test for those that showed significant differences among the 3 groups. Correlations were analyzed using Spearman’s rank correlation coefficient test. Statistical significance was set at *P* < .05. All statistical analyses were performed using EZR (Saitama Medical Center, Jichi Medical University) [27], which is a graphical user interface for R (R Foundation for Statistical Computing). More precisely, it is a modified version of R Commander, designed to add statistical functions frequently used in biostatistics.

## 3 Results

### 3.1 Patient background

Blood samples were drawn from all the patients at 135 points. Patient backgrounds are shown in the Table. The median age was 57 years (37-69 years), and the male-to-female ratio was 1:1. The primary diseases were acute myeloid leukemia in 5 patients, acute lymphoid leukemia in 2 patients, myelofibrosis in 2 patients, myelodysplastic neoplasms in 2 patients, adult T-cell lymphoma/leukemia in 1 patient, mixed-phenotype acute leukemia in 1 patient, and blast phase of chronic myeloid leukemia in 1 patient. Of the 14 patients, 3 underwent transplantation in complete remission (CR), and 11 underwent transplantation in non-CR. The ABO blood types were major mismatches in 3 cases, minor mismatches in 5 cases, major minor mismatches in 1 case, and matches in 5 cases. The transplant source was related peripheral blood in 1 case, unrelated peripheral blood in 6 cases, unrelated bone marrow in 2 cases, and cord blood in 5 cases. Conditioning included fludarabine, melphalan, and busulfan in 13 patients and total body irradiation/cyclophosphamide in 1 patient. Graft-vs-host disease prophylaxis included tacrolimus + short-term methotrexate in 3 patients, tacrolimus + mycophenolate mofetil in 4 patients, and tacrolimus + mycophenolate mofetil + antithymocyte globulin in 7 patients. No grade III to IV graft-vs-host disease, sepsis, hemophagocytic syndrome, or sinusoidal obstruction syndrome/veno-occlusive disease (SOS/VOD) occurred during the observation period.

**Table:** Patients’ characteristics.

UPN	Age	Gender	Underlying disease	Disease state	Transplant type	Donor	Conditioning regimen	GVHD prophylaxis	TA-TMA	
									Probable	Definite
1	54	M	AML	CR1	CB	UR	FLU/MEL/BU	TAC + sMTX	-	-
2	37	F	CML	BP	PB	UR	FLU/MEL/BU	TAC + MMF + ATG	Days 14-42	-
3	60	F	MF	Non-CR	PB	UR	FLU/MEL/BU	TAC + MMF + ATG	Days 7, 42-63	-
4	48	M	AML	Non-CR	CB	UR	FLU/MEL/BU	TAC + MMF	-	-
5	63	F	MF	Non-CR	PB	UR	FLU/MEL/BU	TAC + MMF + ATG	-	-
6	38	M	MDS	Non-CR	CB	UR	FLU/MEL/BU	TAC + sMTX	Day 42	-
7	64	F	AML	Non-CR	PB	R	FLU/MEL/BU	TAC + MMF + ATG	Days 14-28	Days 35-49
8	68	M	AML	Non-CR	PB	UR	FLU/MEL/BU	TAC + MMF + ATG	-	-
9	45	M	ALL	CR1	PB	UR	TBI/CY	TAC + MMF + ATG	Days 14, 35-49	-
10	69	F	ATL	CR1	CB	UR	FLU/MEL/BU	TAC + MMF	Days 7, 14, 35	Days 21, 28
11	69	F	MPAL	Non-CR	PB	UR	FLU/MEL/BU	TAC + MMF + ATG	-	-
12	51	M	AML	Non-CR	CB	UR	FLU/MEL/BU	TAC + MMF	-	-
13	47	M	ALL	Non-CR	BM	UR	FLU/MEL/BU	TAC + MMF	Days 28-42	-
14	65	F	MDS	Non-CR	BM	UR	FLU/MEL/BU	TAC + sMTX	Days 28-42	-

All patients were Japanese.

ALL, acute lymphoid leukemia; AML, acute myeloid leukemia; ATG, antithymocyte globulin; ATL, adult T-cell lymphoma/leukemia; BM, bone marrow; BP, blast phase; BU, busulfan; CB, cord blood; CML, chronic myeloid leukemia; CR, complete remission; CY, cyclophosphamide; F, female; FLU, fludarabine; GVHD, graft-vs-host disease; M, male; MDS, myelodysplastic neoplasms; MEL, melphalan; MF, myelofibrosis; MMF, mycophenolate mofetil; MPAL, mixed-phenotype acute leukemia; PB, peripheral blood; R, related; sMTX, short-term methotrexate; TAC, tacrolimus; TA-TMA, transplant-associated thrombotic microangiopathy; TBI, total body irradiation; UPN, unique patient number; UR, unrelated.

### 3.2 Development of TA-TMA

The transition from probable to definite TMA was observed in 2 of 14 patients (14.3%, unique patient number [UPN]-7 and UPN-10), and 6 of 14 patients (42.9%) presented with probable TMA but did not progress to definite TMA (Table). Of the 135 blood collection points, 5 met the diagnostic criteria for definite TMA, 27 for probable TMA, and 103 for non-TMA.

### 3.3 VWF-associated markers

**Figure 1:**
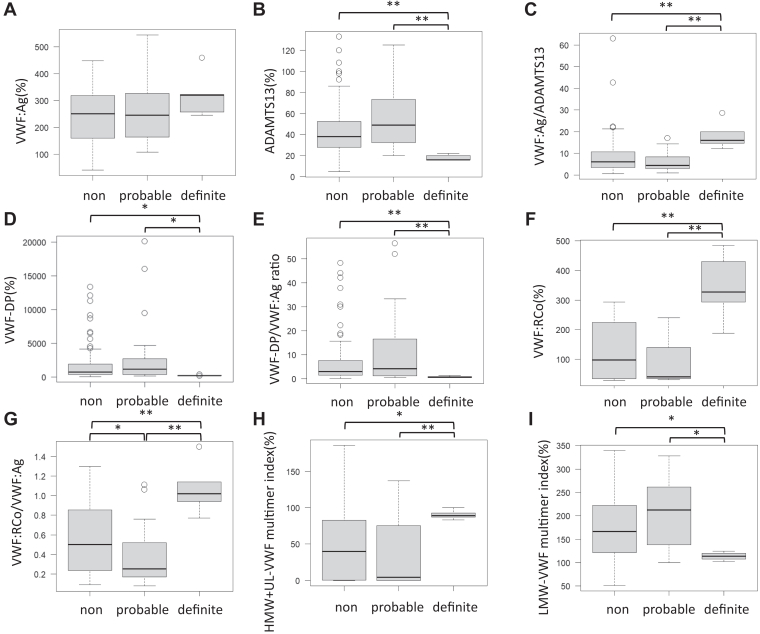
Comparison of von Willebrand factor (VWF)-associated markers in nonthrombotic microangiopathy (TMA), probable TMA, and definite TMA groups. In all 135 blood sampling points, 5 samples were classified as definite TMA, 27 as probable TMA, and 103 as non-TMA. (A) The VWF:antigen (VWF:Ag) level was not significantly different among the 3 groups. (B) ADAMTS13 activity was significantly lower in the definite TMA group than in the non- and probable TMA groups. (C) VWF:Ag/ADAMTS13 activity was significantly higher in the definite TMA group than in the non- and probable TMA groups. (D) The VWF-degradation product (DP) was significantly lower in the definite TMA group than in the non- and probable TMA groups. (E) The VWF-DP/VWF:Ag ratio was significantly lower in the definite TMA group than in the non- and probable TMA groups. (F) The VWF:ristocetin cofactor activity (RCo) was significantly higher in the definite TMA group than in the non- and probable TMA groups. (G) The VWF:RCo/VWF:Ag was significantly different among the 3 groups, with the highest to lowest being the definite, non-, and probable TMA groups. (H) The high-molecular-weight (HMW) + unusually large (UL)-VWF multimer index was significantly higher in the definite TMA group than in the non- and probable TMA groups. (I) The low-molecular-weight (LMW)-VWF multimer index was significantly lower in the definite TMA group than in the non- and probable TMA groups. ∗*P* < .05, ∗∗*P* < .01.

A comparison of VWF:Ag, ADAMTS13 activity, VWF:Ag/ADAMTS13 activity, VWF-DP, VWF-DP/VWF:Ag ratio, VWF:RCo, VWF:RCo/VWF:Ag, HMW + UL-VWF multimer index, and LMW-VWF multimer index in the 3 groups of non-TMA (*n* = 103), probable TMA (*n* = 27), and definite TMA (*n* = 5) patients is shown in Figure 1.

The median VWF:Ag levels were 250% (IQR, 161%-319%), 245% (IQR, 164%-326%), and 320% (IQR, 257%-323%) in the non-TMA, probable TMA, and definite TMA groups, respectively. No significant differences were observed among the 3 groups.

Median ADAMTS13 activity levels were 39% (IQR, 28%-52.5%), 48% (IQR, 32.5%-73.5%), and 16% (IQR, 16%-20%) in the non-TMA, probable TMA, and definite TMA groups, respectively. The value in the definite TMA group was significantly lower than that in the other 2 groups.

The median VWF:Ag/ADAMTS13 activity levels were 6.1 (IQR, 3.4-10.7), 4.4 (IQR, 3.0-8.5), and 16.1 (IQR, 14.7-20) in the non-TMA, probable TMA, and definite TMA groups, respectively. The value in the definite TMA group was significantly higher than that in the other 2 groups.

The median VWF-DP values were 720.0% (IQR, 325.0%-1878.4%), 1162.8% (IQR, 357.8%-2675.2%), and 207.0% (IQR, 198.0%-237.2%) in the non-TMA, probable TMA, and definite TMA groups, respectively.

The median VWF-DP/VWF:Ag ratios were 3.09 (IQR, 1.53-7.69), 4.17 (IQR, 1.26-16.57), and 0.64 (IQR, 0.43-0.97) in the non-TMA, probable TMA, and definite TMA groups respectively. The value in the definite TMA group was significantly lower than that in the other 2 groups.

The median VWF:RCo values were 98% (IQR, 35%-225%), 41% (IQR, 34%-140%), and 327% (IQR, 294%-430%) in the non-TMA, probable TMA, and definite TMA groups, respectively. The value in the definite TMA group was significantly higher than that in the other 2 groups. The VWF:RCo/VWF:Ag differed significantly among the 3 groups: the median values were 0.50 (IQR, 0.24–0.86), 0.25 (IQR, 0.17–0.52), and 1.02 (IQR, 0.94–1.14) for the non-TMA, probable TMA, and definite TMA groups, respectively.

The median HMW + UL-VWF multimer index values were 40.0% (IQR, 0.4%-83.1%), 4.4% (IQR, 0%-75.3%), and 89.5% (IQR, 87.3%-92.8%) in the non-TMA, probable TMA, and definite TMA groups, respectively. The value in the definite TMA group was significantly higher than that in the other 2 groups. The median LMW-VWF multimer index values were 166.4% (IQR, 121.3%-222.5%), 212.8% (IQR, 137.8%-261.5%), and 113.9% (IQR, 107.3%-119.8%) in the non-TMA, probable TMA, and definite TMA groups, respectively. The value in the definite TMA group was significantly lower than that in the other 2 groups.

### 3.4 Relationship between VWF multimer index and VWF:RCo/VWF:Ag and VWF-DP/VWF:Ag ratio

**Figure 2:**
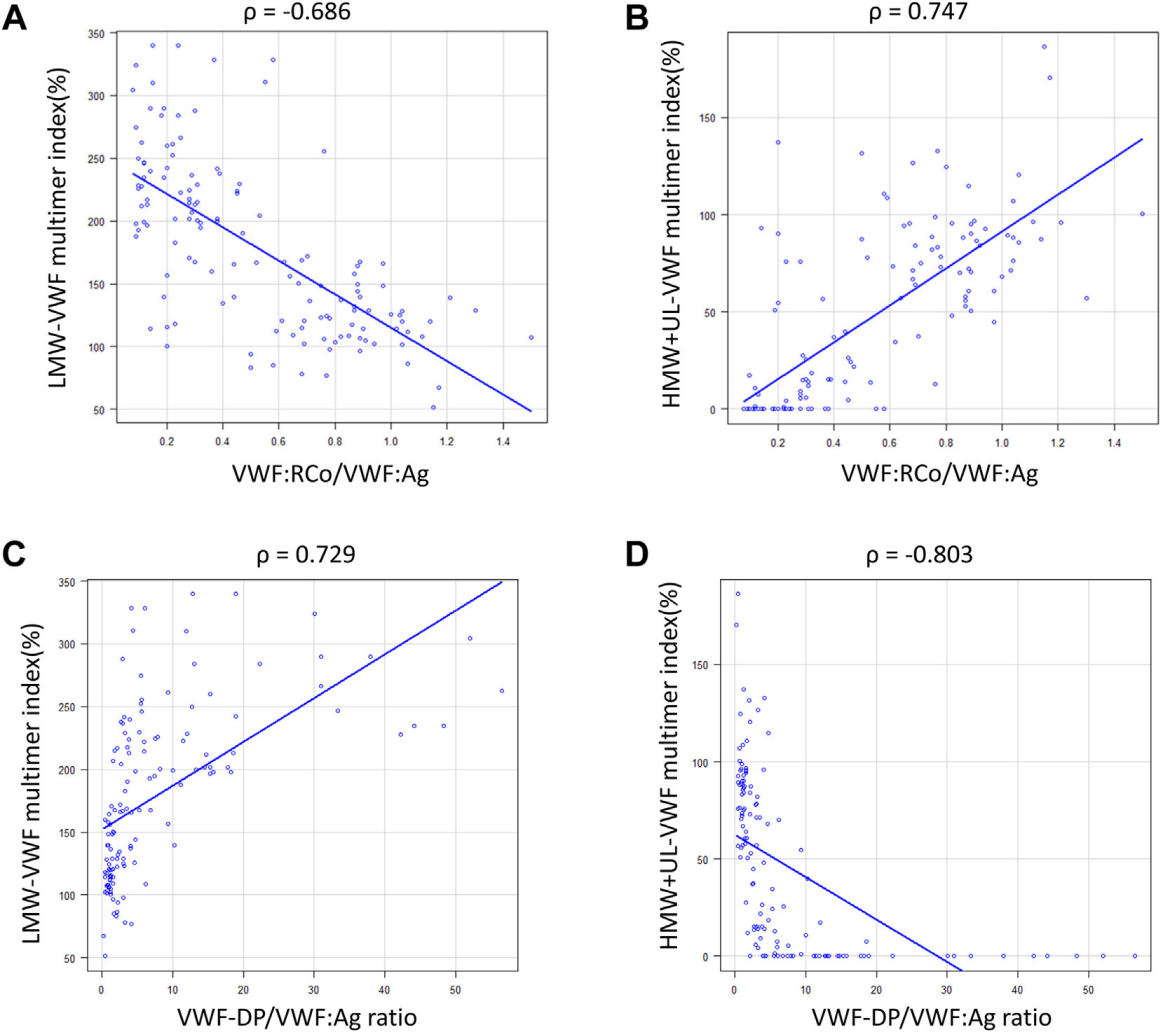
Correlation between von Willebrand factor (VWF) multimer index and VWF:ristocetin cofactor activity/VWF:antigen (VWF:RCo/VWF:Ag), and VWF-degradation product (DP)/VWF:Ag ratio. The Spearman’s rank correlation coefficient test was used. (A) Negative correlation between low-molecular-weight (LMW)-VWF multimer index and VWF:RCo/VWF:Ag, (B) positive correlation between high-molecular-weight (HMW) + unusually large (UL)-VWF multimer index and VWF:RCo/VWF:Ag, (C) positive correlation between LMW-VWF multimer index and VWF-DP/VWF:Ag ratio, and (D) negative correlation between HMW + UL-VWF multimer index and VWF-DP/VWF:Ag ratio are shown (*P* < .01).

A significant negative correlation was observed between the LMW-VWF multimer index and VWF:RCo/VWF:Ag (ρ = −0.686; *P* < .01), as well as between the HMW + UL-VWF multimer index and VWF:RCo/VWF:Ag (ρ = 0.747; *P* < .01; Figure 2). VWF-DP/VWF:Ag and the LMW-VWF multimer index values were significantly correlated (ρ = 0.729; *P* < .01), and significant negative correlation was observed between VWF-DP/VWF:Ag and the HMW + UL-VWF multimer index (ρ = −0.803; *P* < .01). A significant negative correlation (ρ = −0.688; *P* < .01) was observed between VWF:RCo/VWF:Ag and VWF-DP:VWF:Ag.

### 3.5 VWF multimer analysis and diagnosis of TA-TMA

**Figure 3:**
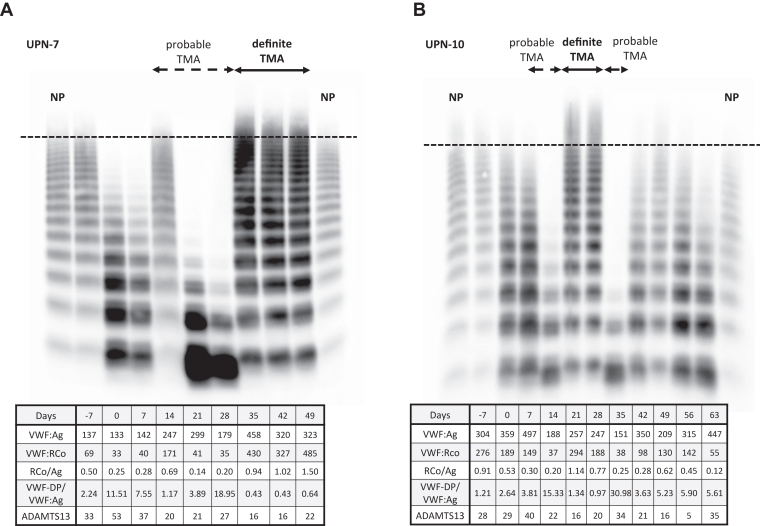
von Willebrand factor (VWF) multimer and VWF-associated markers in 2 patients who developed definite thrombotic microangiopathy (TMA). (A) Data from unique patient number (UPN)-7 are shown. On days 14- to 28, patients met the diagnostic criteria for probable TMA and had high-molecular-weight (HMW)-VWF multimer defects, while on days 35 to 49, an unusually large VWF multimer appeared during definite TMA. The loss of HMW-VWF multimer was thought to be caused by ADAMTS13–induced cleavage of VWF multimer, based on the increased VWF-degradation product (DP)/VWF:antigen (VWF:Ag) ratio. The patient died of multiorgan dysfunction due to transplant-associated TMA on day 49. (B) Data from UPN-10 are shown. Days 7 and 14 met the diagnostic criteria for probable TMA, days 21 and 28 met the diagnostic criteria for definite TMA, and recovery from definite to probable TMA was seen on day 28. HMW-VWF multimer was deficient during probable TMA, and the unusually large VWF multimer appeared during definite TMA. The loss of HMW-VWF multimer was thought to be caused by ADAMTS13 cleavage of the VWF multimer based on the increased VWF-DP/VWF:Ag ratio. Transplant-associated TMA improved in this patient, but she died due to the recurrence of the primary disease. In both cases, there was a point of HMW-VWF multimer loss that did not meet the diagnostic criteria for probable TMA. NP, normal plasma; VWF:RCo, von Willebrand factor ristocetin cofactor activity.

**Figure 4:**
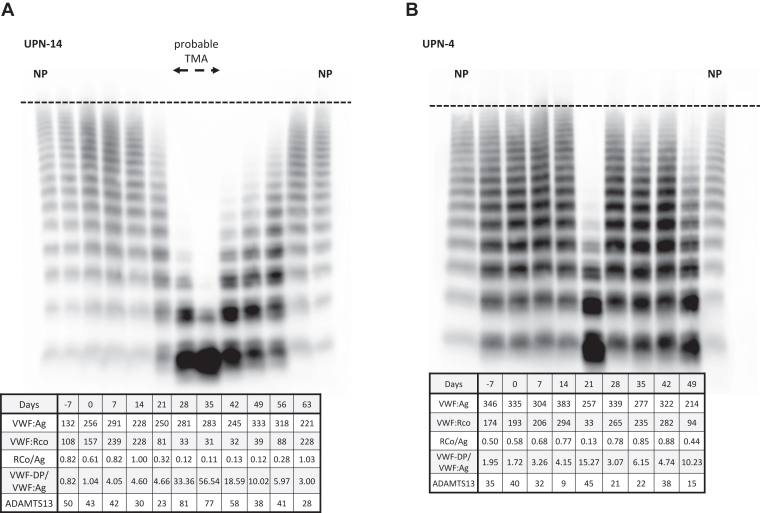
von Willebrand factor (VWF) multimer and VWF-associated markers in probable thrombotic microangiopathy (TMA) and non-TMA patients. (A) Data from unique patient number (UPN)-14 are shown. Days 28 to 35 met the diagnostic criteria for probable TMA and had a deficiency of high-molecular-weight (HMW)-VWF multimer. The loss of the HMW-VWF multimer was due to the cleavage of the VWF multimer by ADAMTS13, based on the increased VWF-degradation product (DP)/VWF:antigen (VWF:Ag) ratio. (B) UPN-4 data are shown. This patient did not develop TMA but died on day 49 due to a recurrence of the primary disease. In both cases, the patient showed a loss of the HMW-VWF multimer during a period that did not meet the diagnostic criteria for probable TMA, which was thought to be caused by ADAMTS13 cleavage of the VWF multimer based on an increased VWF-DP/VWF:Ag ratio. NP, normal plasma; VWF:RCo, von Willebrand factor ristocetin cofactor activity.

VWF multimer analysis and VWF-associated markers for the 2 cases that progressed from probable to definite TMA are shown in Figure 3. Figure 4 shows VWF multimer analysis and VWF-associated markers for the 2 cases. One patient presented with probable TMA but did not develop definite TMA (Figure 4A). The other patient presented with neither probable nor definite TMA (Figure 4B). UL-VWF multimers were present at only 5 points within the definite TMA group.

## 4 Discussion

To our knowledge, this is the first report to investigate the association between TA-TMA onset, VWF-associated markers (VWF:Ag, VWF-DP/VWF:Ag ratio, VWF:RCo, VWF:RCo/VWF:Ag, and ADAMTS13 activity), and VWF multimers over time and in detail. Unlike previous reports [19,20,28,29], our report found that VWF:Ag elevation alone was not a predictive marker for the development of TA-TMA. In addition, VWF:Ag was consistently elevated regardless of the TA-TMA status; VWF:Ag may be more influenced by endothelial damage derived from conditioning chemotherapy, total body irradiation, or calcineurin inhibitors [8] than by TA-TMA. Intriguingly, VWF multimer analysis revealed drastic changes in VWF-related markers in patients with TA-TMA. Upon transition from non-TMA to probable TMA, the HMW-VWF multimer was absent, and VWF:RCo/VWF:Ag was elevated (UPN-7 days 21 and 28; UPN-10 day 14; UPN-14 days 28, 35, and 42). Furthermore, upon transition from probable TMA to definite TMA, the appearance of the UL-VWF multimer and an increase in VWF:RCo/VWF:Ag were observed (UPN-7 days 35, 42, and 49; UPN-10 days 21 and 28). Interestingly, this movement of VWF-associated markers was also observed during recovery from a definite TMA. In other words, during recovery from definite TMA to probable TMA, there was a loss of UL-VWF multimers, an absence of HMW-VWF multimers, and a decrease in VWF:RCo/VWF:Ag; during recovery from probable TMA to non-TMA, there was a reappearance of HMW-VWF multimers and normalization of VWF:RCo/VWF:Ag (UPN-10 days 35 and 42).

Similar VWF multimer behavior has been reported in SOS/VOD [30] caused by damage to sinusoidal endothelial cells [31]. Although TMA and SOS/VOD are not the same disease, in that TMA is caused by vascular endothelial cell injury [32], it is interesting to observe similar VWF multimer behavior in the 2 thrombotic diseases after HSCT. The reason for the loss of the HMW-VWF multimer was not revealed in the SOS/VOD report [30]. VWF-DP, developed in our laboratory, is a measuring system used to quantify VWF cleavage by ADAMTS13, and a high VWF-DP/VWF:Ag ratio indicates increased VWF cleavage by ADAMTS13 [23]. Another disease with dynamic VWF multimer behavior is TTP. In TTP, HMW-VWF multimer is deficient in the acute phase, and HMW-VWF multimer increases immediately before recurrence. HMW-VWF multimer is usually consumed by massive thrombus formation in the acute phase [33,34], which is different from the mechanism of HMW-VWF multimer loss in TA-TMA proposed in our study.

VWF-DP/VWF:Ag was elevated in probable TMA but significantly decreased in definite TMA. HMW-VWF multimer defects after HSCT were derived from the excessive cleavage of the VWF multimer by ADAMTS13.

Our results led to 2 hypotheses regarding the role of VWF multimers in TA-TMA progression. In the first mechanism, microthrombus formation due to vascular endothelial damage causes stenosis of the vascular lumen. This stenosis generates shear stress [35], which stretches the VWF multimeric structure, leading to cleavage by ADAMTS13 [36]. Even in patients without TMA who do not meet the diagnostic criteria for probable or definite TMA, stenosis of the vascular lumen can cause this pathology when microthrombi form due to endothelial damage. This can be explained by the increased VWF-DP/VWF:Ag ratios in the non-TMA and probable TMA groups. The second mechanism is the adhesion of type IV collagen under the basement membrane of endothelial cells to circulating blood due to vascular endothelial damage or vascular disruption [37]. Moreover, VWF multimers are accessible for cleavage by ADAMTS13 when VWF multimers tether to the adhesion site of endothelial cells as a stepping stone. However, these hypotheses fail to explain the mechanism of transition from probable to definite TMA. We speculate that this transition occurs when ADAMTS13 is not sufficiently supplied to cleave VWF multimers. Although it is unclear whether ADAMTS13 is consumptively decreased, definite TMA has significantly lower ADAMTS13 activity than non-TMA, and probable TMA supports this theory.

**Figure 5:**
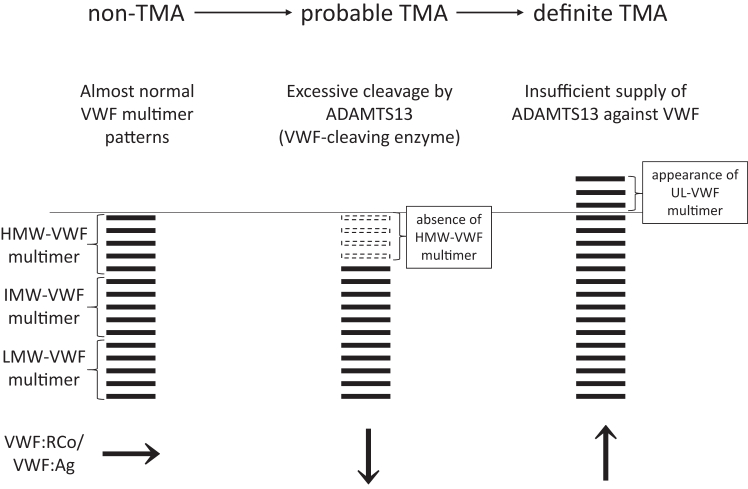
Overview of our findings. In nonthrombotic microangiopathy (TMA), von Willebrand factor (VWF) multimers are almost normal. In probable TMA, there is an absence of high-molecular-weight (HMW)-VWF multimer derived from excessive cleavage by ADAMTS13; VWF:ristocetin cofactor activity/VWF:antigen (VWF:RCo/VWF:Ag) is reduced. In definite TMA, there is an appearance of unusually large (UL)-VWF multimer derived from an insufficient supply of ADAMTS13 against VWF; VWF:RCo/VWF:Ag is increased. IMW, intermediate molecular weight.

When the absence of HMW-VWF multimers occurs because of excessive cleavage by ADAMTS13, a transition from non-TMA to probable TMA may occur. Furthermore, when HMW- or UL-VWF multimers appear in that state, the condition may transition from probable to definite TMA. However, it is difficult to perform rapid VWF multimer analyses and VWF-DP measurements in daily clinical practice. There are commercially available reagents for VWF:Ag and VWF:RCo, which can be measured promptly. In this study, VWF:RCo/VWF:Ag and VWF multimer index were strongly correlated (Figure 2). Importantly, an increase in VWF:RCo/VWF:Ag from this state may be interpreted as a transition from probable to definite TMA. Figure 5 summarizes the overview of our findings and their clinical implications. Measuring VWF:Ag and VWF:RCo is a convenient strategy for estimating the transition of VWF multimers and facilitates recognizing the status of TMA.

This study has several limitations. Although this was a prospective study, it was a single-center study. The number of non-CR cases was large, and there was bias in the donor source and the conditioning regimen. Second, there was a large bias in the number of samples, with 103 non-TMA, 27 probable TMA, and 5 definite TMA samples. Finally, bleeding or thrombotic events were not recorded, and the association between TMA status and thrombotic/hemorrhagic events was not considered. A multicenter prospective validation study, including records of thrombotic/hemorrhagic events in a larger number of patients, should be performed in the future. In addition, complement is important in TA-TMA. In our analysis of VWF-associated markers, citrate plasma was collected and stored; however, for complement analysis, EDTA plasma must be stored at −80 °C or measured immediately after blood collection. Although complement measurement was not possible in this study, it is an important issue to be considered in the future.

Our findings suggest a potential treatment strategy for TA-TMA. There have been reports suggesting that treatment options for TA-TMA include reduction or discontinuation of calcineurin inhibitors that exacerbate vascular endothelial damage, control of infection and graft-vs-host disease [4], and recombinant thrombomodulin [38] and fresh frozen plasma infusion [30]. However, the efficacy of plasma exchange remains controversial [39]. Completion of TA-TMA leads to organ ischemia, multiorgan failure, and death. To prevent probable TMA from turning into definite TMA and to prevent the transition from definite TMA to organ ischemia and multiorgan failure, treatment that does not induce UL-VWF multimers or eliminate or degrade UL-VWF multimers may be an effective option for definite TMA. Recombinant ADAMTS13 [40] may improve the pathophysiology of definite TMA by degrading the UL-VWF multimer but may not improve the pathophysiology of probable TMA and may induce bleeding due to HMW-VWF multimer deficiency in probable TMA. Repeated fresh frozen plasma infusions supplemented with ADAMTS13 and appropriately sized VWF multimers may be the best TA-TMA therapy currently available. In addition, as a measurement of the clinical response, a decrease in VWF:RCo/VWF:Ag may be used as an indicator of improvement from definite TMA to probable TMA, and an increase in VWF:RCo/VWF:Ag may be used as an indicator of improvement from probable TMA to non-TMA.

## 5 Conclusions

Despite a small sample size in a single institute, we found a dynamic change in VWF multimer analysis from the loss of HMW-VWF multimers to the appearance of UL-VWF multimers during the transition from probable to definite TMA, suggesting that excessive cleavage of VWF multimers by ADAMTS13 may predict the progression of TA-TMA. Based on the VWF multimer analysis, it is desirable to develop safe TA-TMA therapy from the viewpoint of both hemorrhage and thrombosis.
