# Supplementary material for: Excessive cleavage of von Willebrand factor multimers by ADAMTS13 may predict the progression of transplant-associated thrombotic microangiopathy
Source: Res Pract Thromb Haemost. 2024 Jul 22;8(5):102517. doi: 10.1016/j.rpth.2024.102517 (PMC11378204; doi:10.1016/j.rpth.2024.102517)

**Excessive cleavage of von Willebrand factor multimers by ADAMTS13 may predict the progression of transplant-associated thrombotic microangiopathy**

Shinya Yamada^a,b,c,^ Kazuya Sakai^b^, Masayuki Kubo^b,d,^ Hirokazu Okumura^c^, Hidesaku Asakura^a^, Toshihiro Miyamoto^a^, Masanori Matsumoto^b,d^*

^a^ Department of Hematology, Kanazawa University, Kanazawa City, Ishikawa, Japan

^b^ Department of Blood Transfusion Medicine, Nara Medical University, Kashihara City, Nara, Japan

^c^ Department of Hematology, Toyama Prefectural Central Hospital, Toyama City, Toyama, Japan

^d^ Department of Hematology, Nara Medical University, Kashihara City, Nara, Japan

**Supplementary Figure S1. Quantification of VWF multimer index**

VWF multimer analysis was performed as previously described [14] with some modifications [24,25]. VWF multimer bands were classified into three parts: LMW (the five fastest-moving smallest multimers), IMW (multimers 6-10), and HMW (multimers > 10) + UL (VWF multimers not present in normal plasma). The center of each lane was scanned using ImageJ software (National Institutes of Health, Bethesda, MD, USA) for densitometric analysis. The VWF multimer ratio was calculated by dividing the intensity of each part of the normal or patient plasma by the total VWF area intensity. The VWF multimer index was calculated by dividing the VWF multimer ratio of each part of the patient’s plasma by the VWF multimer ratio of each part of the normal plasma.

VWF, von Willebrand factor; LMW, low molecular weight; IMW, intermediate molecular weight; HMW, high molecular weight; UL, unusually large


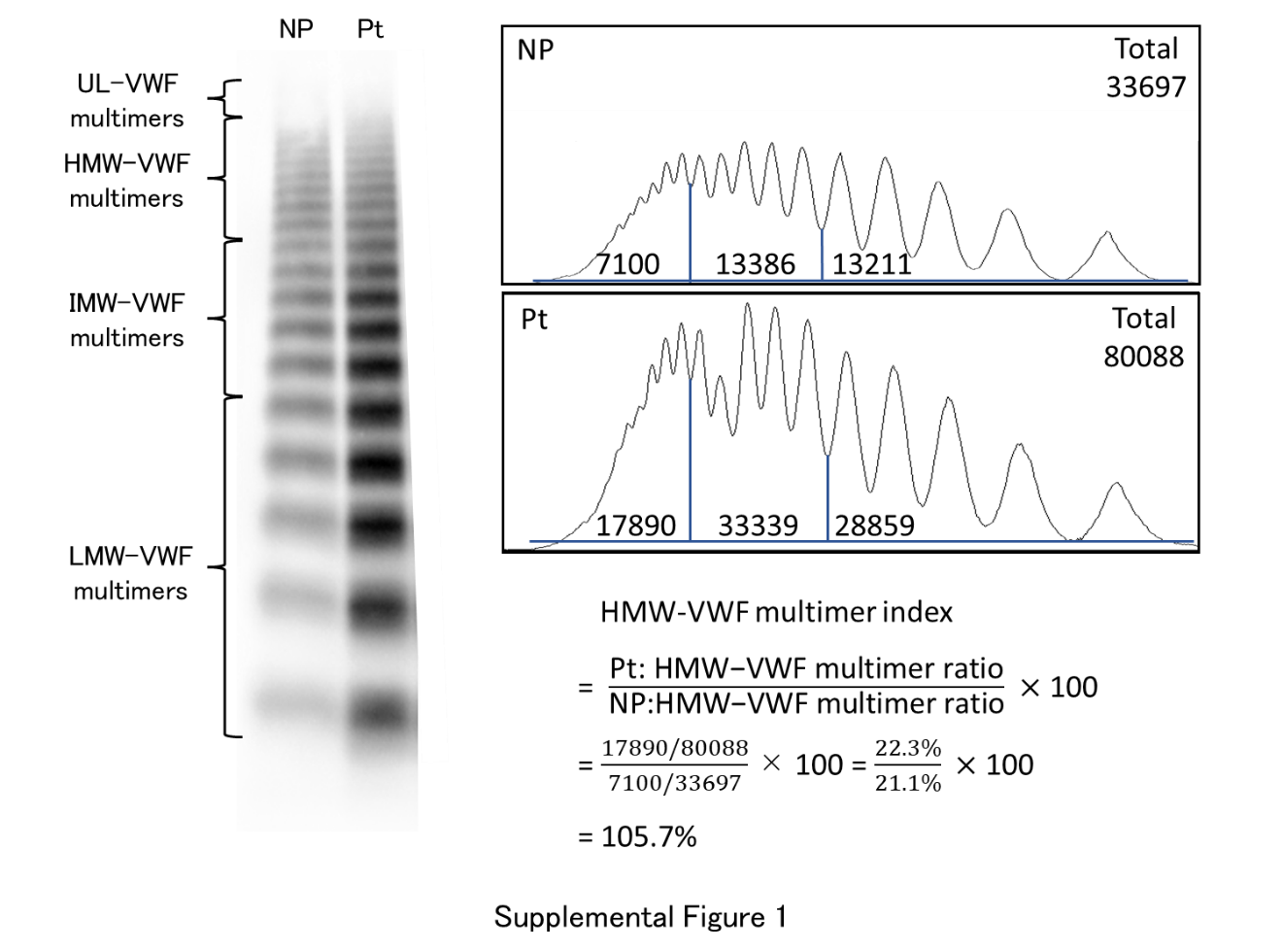

Supplement: Supplemental Fig.1 [file mmc1.docx]
